# Peripheral innate immunophenotype in neurodegenerative disease: blood-based profiles and links to survival

**DOI:** 10.1038/s41380-024-02809-w

**Published:** 2024-10-29

**Authors:** Alexandra Strauss, Peter Swann, Stacey L. Kigar, Rafailia Christou, Natalia Savinykh Yarkoni, Lorinda Turner, Alexander G. Murley, Leonidas Chouliaras, Noah Shapiro, Nicholas J. Ashton, George Savulich, W. Richard Bevan-Jones, Ajenthan Surendranthan, Kaj Blennow, Henrik Zetterberg, John T. O’Brien, James B. Rowe, Maura Malpetti

**Affiliations:** 1 University of Cambridge Department of Clinical Neurosciences and Cambridge University Hospitals NHS Trust, Cambridge, UK; 2 Department of Psychiatry, University of Cambridge, Cambridge, UK; 3 Department of Medicine, University Cambridge, Cambridge, UK; 4 Department of Psychiatry and Neurochemistry, University of Gothenburg, Gothenburg, Sweden; 5 Banner Alzheimer’s Institute and University of Arizona, Phoenix, AZ USA; 6 Banner Sun Health Research Institute, Sun City, AZ USA; 7 Clinical Neurochemistry Laboratory, Sahlgrenska University Hospital, Mölndal, Sweden; 8 Department of Neurodegenerative Disease, UCL Institute of Neurology, Queen Square, London, UK; 9 UK Dementia Research Institute at UCL, London, UK; 10 Hong Kong Center for Neurodegenerative Diseases, Clear Water Bay, Hong Kong, China; 11 Wisconsin Alzheimer’s Disease Research Center, University of Wisconsin School of Medicine and Public Health, University of Wisconsin-Madison, Madison, WI USA; 12 Medical Research Council Cognition and Brain Sciences Unit, Cambridge, UK; 13 UK Dementia Research Institute at University of Cambridge, Cambridge, UK

**Keywords:** Neuroscience, Prognostic markers

## Abstract

The innate immune system plays an integral role in the progression of many neurodegenerative diseases. In addition to central innate immune cells (e.g., microglia), peripheral innate immune cells (e.g., blood monocytes, natural killer cells, and dendritic cells) may also differ in these conditions. However, the characterization of peripheral innate immune cell types across different neurodegenerative diseases remains incomplete. This study aimed to characterize peripheral innate immune profiles using flow cytometry for immunophenotyping of peripheral blood mononuclear cells in n = 148 people with Alzheimer’s disease (AD), frontotemporal dementia (FTD), corticobasal syndrome (CBS), progressive supranuclear palsy (PSP), Lewy body dementia (LBD) as compared to n = 37 healthy controls. To compare groups, we used multivariate dissimilarity analysis and principal component analysis across 19 innate immune cell types. We identified pro-inflammatory profiles that significantly differ between patients with all-cause dementia and healthy controls, with some significant differences between patient groups. Regression analysis confirmed that time to death following the blood test correlated with the individuals’ immune profile weighting, positively to TREM2+ and non-classical monocytes and negatively to classical monocytes. Taken together, these results describe transdiagnostic peripheral immune profiles and highlight the link between prognosis and the monocyte cellular subdivision and function (as measured by surface protein expression). The results suggest that blood-derived innate immune profiles can inform sub-populations of cells relevant for specific neurodegenerative diseases that are significantly linked to accelerated disease progression and worse survival outcomes across diagnoses. Blood-based innate immune profiles may contribute to enhanced precision medicine approaches in dementia, helping to identify and monitor therapeutic targets and stratify patients for candidate immunotherapies.

## Introduction

Despite the clinical and pathological heterogeneity characterizing the main dementias, dysregulation of the innate immune system is identified as a common feature in all neurodegenerative diseases [1]. Centrally, activation of cerebral microglia is reported for Alzheimer’s disease (AD) and its prodromal mild cognitive impairment (MCI), Lewy body dementia (LBD) including Parkinson’s disease dementia (PDD) and dementia with Lewy bodies (DLB), and the syndromes associated with frontotemporal lobar degeneration (FTLD). The latter includes the behavioural variant of frontotemporal dementia (bvFTD), non-fluent (nfPPA) and semantic (svPPA) variants of primary progressive aphasia, corticobasal syndrome (CBS), and progressive supranuclear palsy (PSP). Moreover, genome-wide association studies link mutations in genes coding for proteins related to the immune system to the development of multiple neurodegenerative conditions [2–7]. For example, mutations in the gene for triggering receptor expressed on myeloid cells 2 (TREM2, encoding a receptor on monocytes and microglia) may cause FTLD-syndromes and AD, with an effect size comparable to the immune-related APOE4 variant [2, 8]. Such genomic associations are complemented by evidence of abnormalities in central and peripheral innate immune systems in neurodegenerative diseases [9].

Most evidence describing abnormal innate immunity in dementia to date concerns cerebral cells. Specifically, microglial activation in the central nervous system is implicated in many forms of dementia [10–16]. For example, human post mortem studies report activated microglia in association with the severity of amyloid and tau pathology in AD, FTLD-syndromes and LBD [11, 12, 17, 18]. However, post mortem studies are not well suited to characterize microglial changes in early disease stages. Instead, overexpression of the translocator protein 18 kDa (TSPO), overexpressed in activated microglia, can be detected by positron emission tomography (PET). Microglial activation presents early in people living with many types of dementia and predicts the rate of cognitive decline [13–16, 19]. While TSPO PET is a powerful, informative tool to visualize cerebral innate immune activation, it is expensive and not readily scalable. Alternatively, peripheral blood-based markers of immune dysregulation are to be potentially scalable and repeatable over time.

Cells of the innate immune system— including monocytes, dendritic cells, and natural killer (NK) cells – rapidly and non-specifically initiate an immune response upon detection of pathogens or cellular damage [20]. While this process is beneficial in the short-term to eliminate harmful stimuli, chronic or dysregulated activation can lead to disease. The peripheral immune system may interact with the central nervous system indirectly via chemokine and cytokine signalling or directly via infiltration into and meningeal surveillance of the parenchyma [21–27]. Elevated pro-inflammatory cytokine concentrations are reported in post mortem brain tissue and cerebrospinal fluid (CSF) in people with AD, LBD, and FTLD-syndromes [28–31]; while peripheral infections can exacerbate the neuroinflammatory environment and accelerate cognitive decline [26, 32]. Taken together, evidence implicates primary effector cells of the peripheral innate immune system in the pathogenesis of neurodegenerative diseases.

Monocytes, NK cells, and dendritic cells are each implicated in the pathogenesis of dementias. For example, TREM2 is expressed on the surface of peripheral monocytes [33] and may be a marker of monocyte recruitment in AD [27]. Elevated concentration of CSF soluble TREM2 (sTREM2), cleaved from the membranes of microglia or monocytes, is linked to disease progress cognitive decline in AD [34] and FTLD [11]. Beyond TREM2, CSF concentrations of chemokine motif ligand 2 (CCL2), mediating the chemotaxis of monocytes, are also predictive of cognitive decline in AD [35]. Similarly, abnormal activation patterns of NK cells occur with AD [36] and Parkinson’s disease (closely related to DLB and PDD) [37]. Finally, myeloid dendritic cell frequency is reduced in people with AD and Parkinson’s disease [38–40]. Overall, evidence involving monocytes, NK cells, and dendritic cells supports a more integrative neurodegenerative disease processes than initially appreciated.

While the mechanisms of action of peripheral immune cells remain unclear, they are nonetheless more readily accessible to quantify, compared to central innate immune cells, via phlebotomy. The identification of innate cell types in the periphery of people with dementia may clarify links between innate immunity and neurodegenerative disease, and yield clinically relevant, blood-based biomarkers to support target identification and patient stratification for immune-targeting therapies. However, current immunophenotyping data is limited to few diagnoses of dementia and remains incomplete in cellular subtype characterization [38, 39, 41–43]. To meet the needs for diagnostic, prognostic and trialist use, there is a pressing need for improved profiling of peripheral innate immunity in multiple neurodegenerative disorders.

This study therefore aims to characterize innate immune profiles in blood of people with diverse neurodegenerative dementias, including AD, FTLD-syndromes and LBD using Flow Cytometry- a well-established method of identifying cells via their unique expression of activation and lineage markers on the cell surface. We test the hypothesis that innate immune cell profiles are abnormal in people with any of these disorders, with at least partial commonality in the immune profile for the neurodegenerative disorders investigated here (i.e. a transdiagnostic abnormality). Given the multivariate nature of immune activation, we use data driven approaches to reduce dimensionality (complexity) and test for (dis)-similarity between the clinical diagnostic groups. We then highlight clinical relevance by the correlation or multivariate immune profiles with time to death.

## Materials and methods

### Participants

Patients (*n* = 148) were recruited from clinics for cognitive and movement disorders at the Cambridge University Hospitals NHS Trust as well as collaborating regional psychiatry and neurology services.

We included participants with a clinical diagnosis that met the standardised criteria of MCI/AD [44, 45] (*n* = 24; 5 MCI and 19 AD), probable or possible PSP (*n* = 54, predominantly Richardson’s syndrome) [46], CBS [47] (*n* = 23), FTD [48, 49] (*n* = 18; 12 bvFTD and 6 PPA), PDD (*n* = 3), or DLB (*n* = 26) [50]. In a sub-group of participants (*n* = 34), PET and/or CSF markers for amyloid were obtained to confirm the presence or absence of β-amyloid (interpreted as AD pathology) in conditions with weak clinicopathological correlations and either high likelihood of a significant fraction with AD as main or co-pathology (CBS and DLB) or a high likelihood of clinical diagnostic false positives (amnestic MCI). Of the CBS patients, *n* = 12 underwent amyloid PET, which confirmed amyloid positivity in *n* = 4. Of the MCI patients, all were confirmed amyloid-positive with PET imaging with the Pittsburgh compound B (PiB tracer at a cut-off of 19 centiloids [51]) and/or CSF Alzheimer’s biomarkers at lumbar puncture (Amyloid-beta 1-42/40 ratio < 0.065 as recommended laboratory threshold from University College London Hospitals reference Laboratory [52]). Of the DLB cohort, *n* = 6 out of 17 patients resulted amyloid-positive at PiB PET. We also included *n* = 37 healthy controls given MMSE > 26/30, absence of memory symptoms, no signs of dementia, or any other significant medical illnesses [53]. Exclusion criteria for both patient and healthy control cohorts included recent or current acute infection, major concurrent psychiatric illness, other severe physical illness, or a history of other significant neurological illness and/or autoimmune conditions.

All participants underwent baseline clinical and neuropsychological assessment, including the revised Addenbrooke’s Cognitive Examination (ACE-R, 0-100 points) and mini-mental state examination (MMSE). The ACE-R is subdivided into five domains: Attention and Orientation (18/100), Memory (26/100), Fluency (14/100), Language (26/100), and Visuospatial abilities (16/100). In a subset of participants, an informant completed the 8 Item Clinical Dementia Rating (CDR) FTLD NACC (Table 1.) Survival data were collected for all up to and including the 17th of October 2023 (the census date).

**Table 1:** Demographic and clinical summaries for patients and controls.

	Control	AD	CBS	LBD	FTD	PSP	Group difference
*n*	37	24	23	29	18	54	—
Sex, F/M	14/23	9/15	17/6	4/25	6/12	26/28	Chi Squared = 20.31, *p* < 0.001^a^
Age, years, Mean (SD)	69 (7)	72 (7)	69 (9)	74 (7)	69 (8)	72 (7)	ns
MMSE, Mean (SD)	29 (1)	23(5)	25(5)	25(4)	21(9)	27 (4)	H = 49.2, df = 5, *p* < 0.001^a^
ACE-R, Mean (SD)	96 (3)	67 (16)	75 (18)	72 (15)	69 (26)	81 (13)	H = 76.8, df = 5, *p* < 0.001^a^
CDR Sum of Boxes, Mean (SD) (*n* = 127)	0.038 (0.139)	5.12 (2.75)	5.83 (4.14)	8.45 (4.30)	12.2 (5.88)	7.29 (5.06)	H = 46.1, df = 5, *p* < 0.001^a^
*n* Deceased by Census Date	0	2	2	10	8	20	—
*n* Biomarkers Collected	27	17	16	22	12	28	
ptau 217, pg/mL, Mean (SD)	0.331 (0.221)	0.924 (0.339)	0.667 (0.631)	0.666 (0.395)	0.412 (0.201)	0.456 (0.383)	H = 41.2, df = 5, *p* < 0.001^a^
Nfl, pg/mL, Mean (SD)	18.33 (6.75)	32.5 (13.91)	57.133 (41.31)	34.223 (18.65)	58.367 (26.41)	51.272 (27.634)	H = 50.6, df = 5, *p* < 0.001^a^
GFAP, pg/mL, Mean (SD)	107.27 (54.62)	192.8 (105.32)	238.48 (153.67)	161.2 (95.308)	174.50 (102.92)	164.99 (92.064)	H = 18.3, df = 5, *p* = 0.002^a^
AB40/AB42, pg/mL, Mean (SD)	16.98 (2.703)	19.6 (2.38)	17.695 (2.66)	18.721 (5.029)	16.288 (2.21)	19.643 (5.32)	H = 16.0, df= 5, *p* = 0.007^a^

Differences in sex were evaluated using Chi Squared test. Differences in cognitive/clinical test scores and biomarkers were evaluated using Kruskal–Wallace Tests followed by Dunn’s Post hoc analysis giving H.

^a^indicates statistical significance between groups at *p* < 0.05.

*AD* Alzheimer’s disease, *CBS* corticobasal syndrome, *LBD* Lewy body dementia, *FTD* frontotemporal dementia, *PSP* progressive supranuclear palsy, *AB* Amyloid beta, *p-tau* phosphorylated tau, *NfL* Neurofilament light chain, *GFAP* Glial fibrillary acidic protein.

### Ethics approval and consent to participate

Participants with mental capacity gave their written informed consent to take part in the study according to the Declaration of Helsinki. For those who lacked capacity, their participation followed the consultee process in accordance with the UK law. The research protocols were approved by the National Research Ethics Service’s East of England Cambridge Central Committee.

### Blood collection and flow cytometry analyses

At baseline, 18 ml of blood was drawn in EDTA and analysed at the NIHR Cambridge Cell Immunophenotyping Hub. Wherever possible, samples were processed within 2 h (over 98% of samples). Blood was layered onto sterile Ficoll (Cytiva, Cat#: 17144003) for peripheral blood mononuclear cell (PBMC) isolation by a technician blind to group status. Two aliquots containing ~1 × 10^6^ PBMCs were stained using the antibody cocktail shown in Supplementary Table S1, using either TREM2 or a matched isotype control. At the end of staining, cells were washed, and the data were acquired on live (i.e., non-fixed) cells with a BD LSR Fortessa instrument.

Cell classifications were determined via manual gating in FlowJo (BD) by individuals blind to participant group according to standards recommended for the Human Immunology Project [54]. Briefly, monocytes were identified as HLA-DR+/CD14+; populations were further stratified based on CD16 and CD14 positivity where CD16-/CD14hi monocytes were labelled as classical, CD16+/CD14+ monocytes labelled as intermediate, and CD16+/CD14lo monocytes labelled as nonclassical. Each monocyte subpopulation was then gated to discern CCR5+, CCR2+, and TREM2+ staining. Dendritic cells (DCs) were identified as HLA-DR+/CD14- and further stratified based on CD123 and CD11c staining: CD123+/CD11c+ cells were double positive (DC+/+), CD123+/CD11c- were plasmacytoid DCs, CD123-/CD11c+ were myeloid DCs, and CD123-/CD11c- were double negative (DC-/-). NK cells were identified as CD56+ and further subdivided into two groups as either CD16+ (which were CD56lo) or CD16- (which were CD56hi). The full gating strategy is found in Supplementary Fig. S2. Interrater validation studies showed excellent concordance between different evaluators (Supplementary Fig. S3; Supplementary Table S2).

### Plasma dementia-related biomarkers

In a sub-cohort of 122 participants (Control = 27, AD = 17, CBS = 16, FTD = 12, LBD = 22, PSP = 28), plasma samples were stored at −70 °C for further analyses at the Clinical Neurochemistry Laboratory in Mölndal (Sweden). Plasma samples were thawed on wet ice, centrifuged at 500× g for 5 min at 4 °C. Calibrators (neat) and samples (plasma: 1:4 dilution) were measured in duplicates. The plasma assays performed were the Quanterix Simoa Human Neurology 4-Plex E assay (measuring Aβ40, Aβ42, GFAP and NfL, Quanterix, Billerica, MA) and the p-tau217 ALZpath assay measuring p-tau217 of the human tau protein associated with AD, as previously described [55]. Plasma samples were analysed at the same time using the same batch of reagents. A four-parameter logistic curve fit data reduction method was used to generate a calibration curve. Two control samples of known concentration of the protein of interest (high-control and low-control) were included as quality control. Intra-assay coefficients of variation were below 10%.

### Statistical analysis

Analyses were performed using R (Version 2023.03.0 + 386). Non-parametric tests were used for all pairwise comparisons given non-normal data distributions. For inference, *p*-values were corrected for multiple comparisons using the Benjamini-Hochberg procedure to control the false discovery rate (FDR) and considered significant with threshold *p*-FDR < 0.05. For transparency and explorative analyses, uncorrected *p*-values are also reported. Statistical analysis was carried out in three steps.

First, to understand similarity of innate immune cell profiles between diagnoses and controls, absolute cell numbers from FlowJo were normalized to standardize across minimum and maximum absolute cell count ranges. These normalized cell counts were averaged within each diagnosis to yield an average, normalized 19-cell vector per diagnosis. To evaluate dissimilarity between diagnoses, Euclidean distance was calculated on the normalized data based on a 19-cell vector for all diagnoses followed by single linkage hierarchical clustering.

Second, a Principal Component Analysis (PCA) was applied on the 19 innate immune cell classes. This reduced the dimensionality of our dataset to minimize multiple comparisons, identifying a limited number of components that best explain the data variance. The PCA was computed using cell counts calculated as a proportion of their parent population across all cell types (Supplementary Fig. S1). Local Outlier Factor (LOF) analysis with a hyperparameter of *k* = 10 identified 2 participants as outliers following PCA computation (LOF = 3) [56]. These outliers were excluded before re-computing the PCA. We retained 3 components based on eigenvalues > 1, explained variance > 10%, and application of the visual “elbow method” from the scree plot for further analyses [57, 58] (Supplementary Fig. S4). Individual participant loadings in each of the 3 selected components were included in group-comparison analyses using Mann–Whitney U tests to compare median values between patients and controls, and Kruskal–Wallace tests to compare across patient groups followed by Dunn’s post hoc analysis, where applicable.

Finally, we tested for associations between the individual participant loadings and clinical outcome. Individual component loadings were individually evaluated as a predictor of NfL, p-tau217, Aβ40/Aβ42, GFAP, and ACE-R total scores using a linear regression, controlling for age and sex. Similarly, we investigated each principal component loading in relationship to years of survival following blood draw using a linear model, including age, sex, and biomarkers as covariates.

## Results

Participant summary clinical characteristics are in Table 1. There were 76 female and 109 male participants. Most groups, apart from CBS, had more males than females. As expected, control participants scored significantly higher on the ACE-R examination than each patient group (*p* < 0.001 for all comparisons); while people with AD had significantly lower ACE-R total scores than people with PSP (*p* = 0.002).

### Dissimilarity analysis

**Fig. 1:**
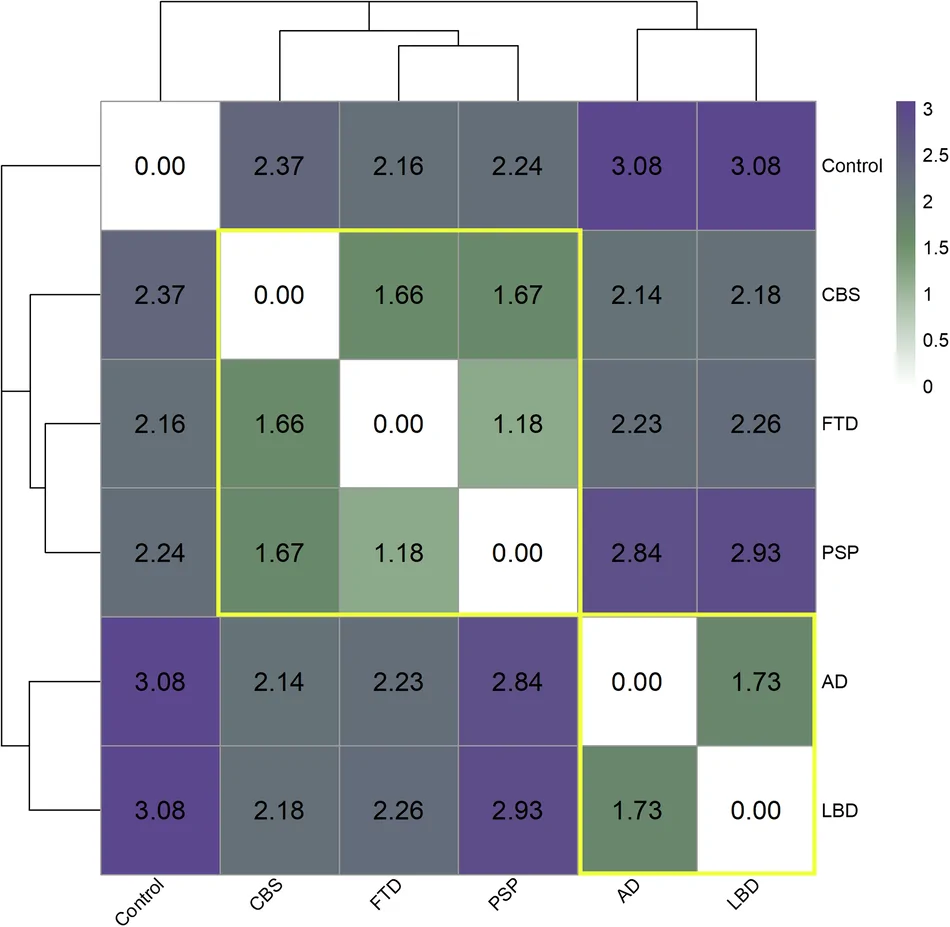
Euclidean Distance dissimilarity analysis and hierarchical clustering. Colours and values represent the Euclidean distance calculated from a 19-cell vector averaged across each group. The value for the Euclidean distance is relative to the dataset, thus the number displayed represents the length of the line segment between groups relative to the total group distance. Darker colours and larger Euclidean distance values indicate greater dissimilarity between 19-cell immune profiles across diagnostic groups, while lighter colours indicate relative similarity. The dendrogram represents single linkage hierarchical clustering. Note that all patients separate from controls initially and that the group of syndromes associated with frontotemporal lobar degeneration are similar to each other (PSP, FTD, and CBS), in contrast to Alzheimer’s disease (AD) and Lewy Body dementia (LBD). These relative similarities are highlighted in yellow.

Figure 1 shows dissimilarity values of the 19-cell vector measured by Euclidean distance between groups (See Supplementary Figs S5 and S6, and Supplementary Tables S7 and S8, for individual cell pairwise comparisons). Hierarchical clustering via single linkage represented in the dendrogram summarizes the group-wise differences in the pattern (rather than magnitude) of immune profiles. Maximal relative distance was found between controls and all patient groups as evidenced by the distinct cluster or branch from the dendrogram. The smallest distance (i.e. most similarity) was identified within PSP and FTD groups, while PSP, CBS and FTD groups were more similar to each other than to AD/LBD. Importantly, the distance value between CBS and AD (2.14) and LBD (2.18) are smaller than the distance between AD, LBD, and other FTLD conditions.

### Principal Component Analysis

**Fig. 2:**
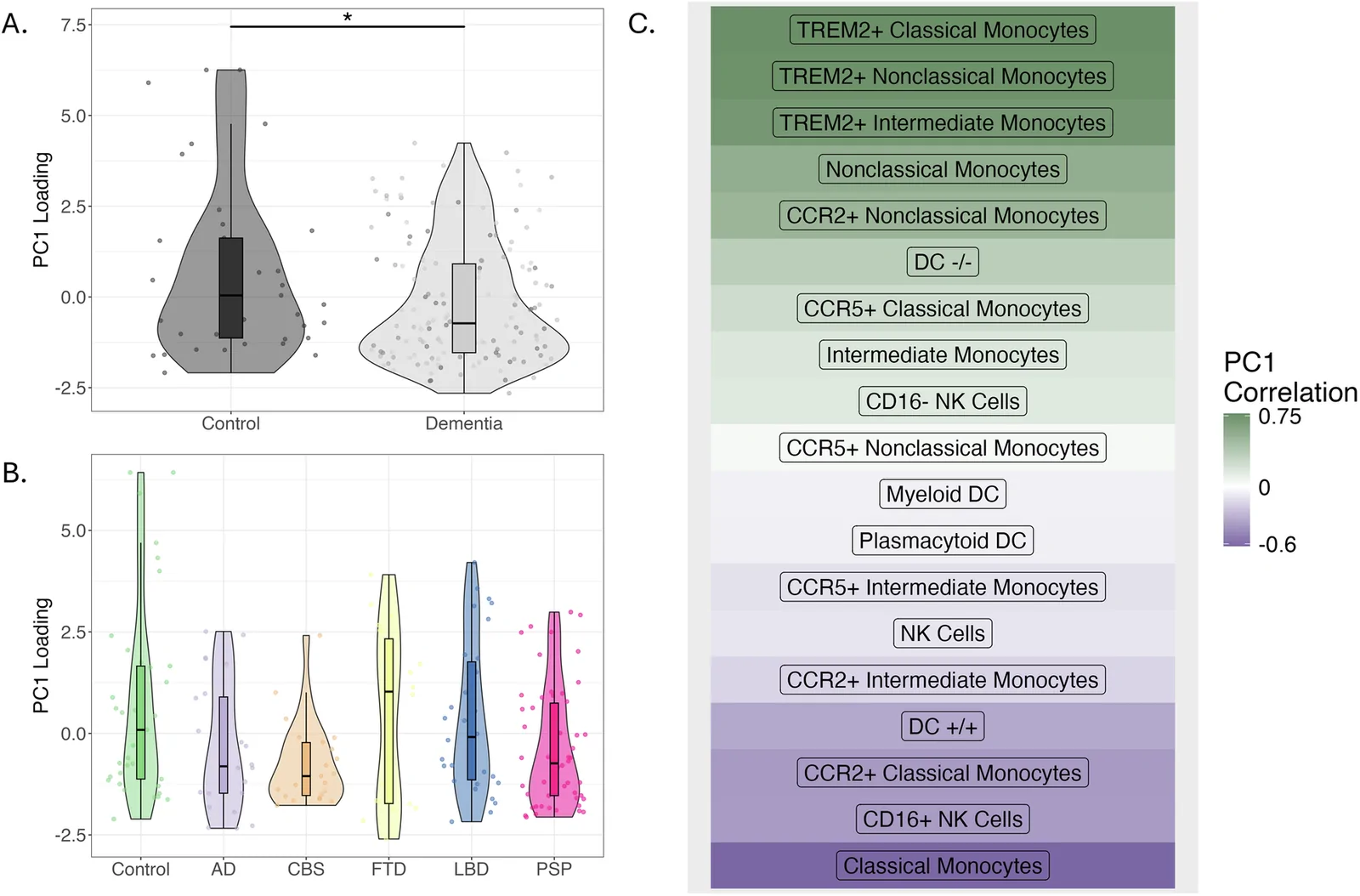
Immune profile loading onto TREM2+ monocytes and nonclassical monocytes (positively) and classical Monocytes (negatively). **A** Median individual loading onto PC1 between controls and all-cause dementia patients (W = 3351, *p* = 0.02). **B** Median individual loading onto PC1 for each diagnostic group. Note the absence of significant differences between patient groups. **C** Correlations of each cell type with PC1. Darker colours indicate stronger positive and negative correlations to PC1. Mann–Whitney U and Kruskal–Wallace tests were used to compare medians. Results were considered significant at *p* <0.05 indicated with *.

**Fig. 3:**
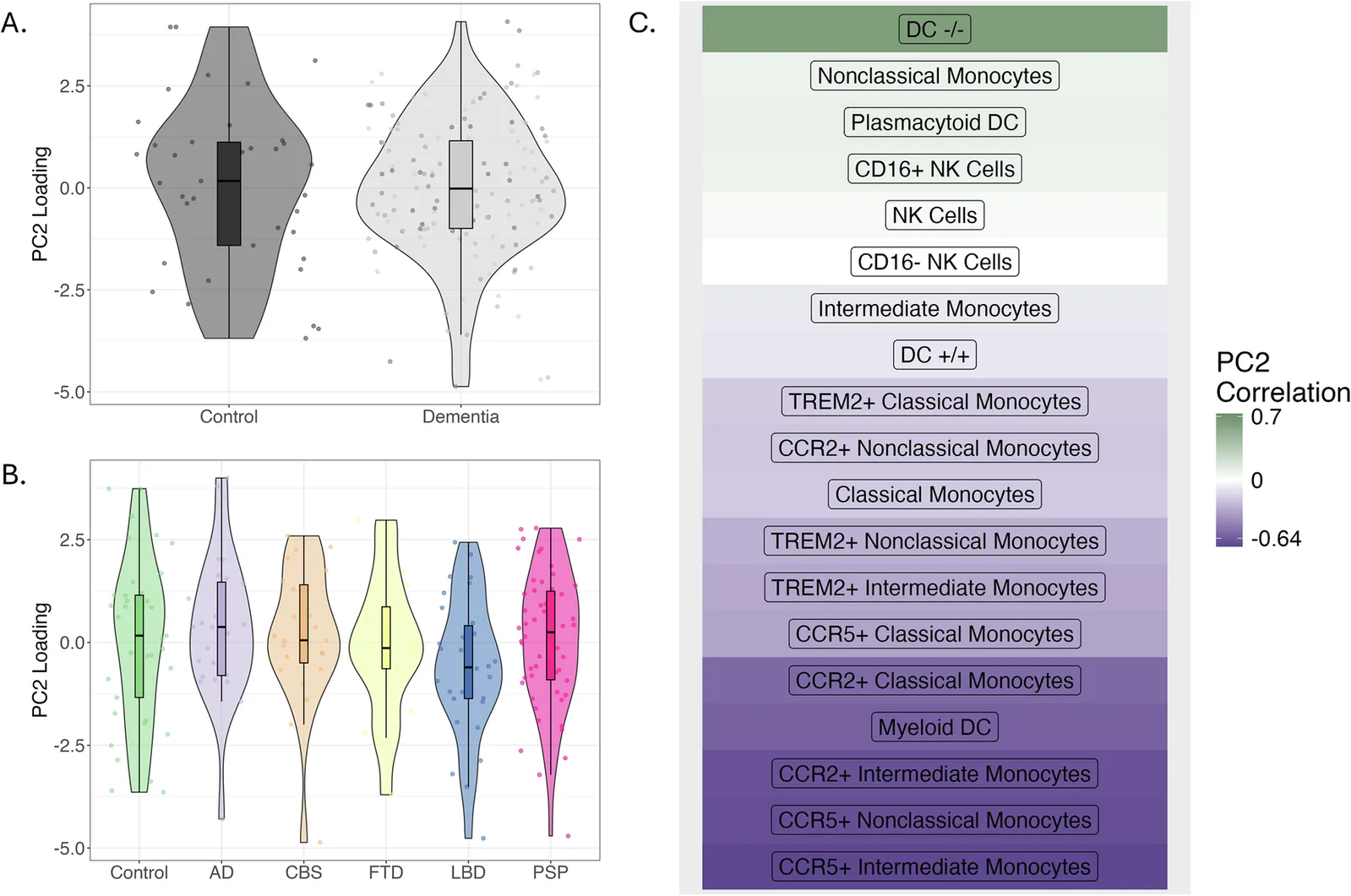
Immune profile loading onto DC-/- cells (positively) and CCR5+ monocytes (negatively). **A** Median individual loading onto PC2 between controls and all-cause dementia patients. **B** Median individual loading onto PC2 for each group. **C** Correlations of each cell type with PC2. Darker colours indicate stronger positive and negative correlations to PC3. Mann–Whitney U and Kruskal–Wallace tests were used to compare medians. Results were considered significant at p < 0.05.

**Fig. 4:**
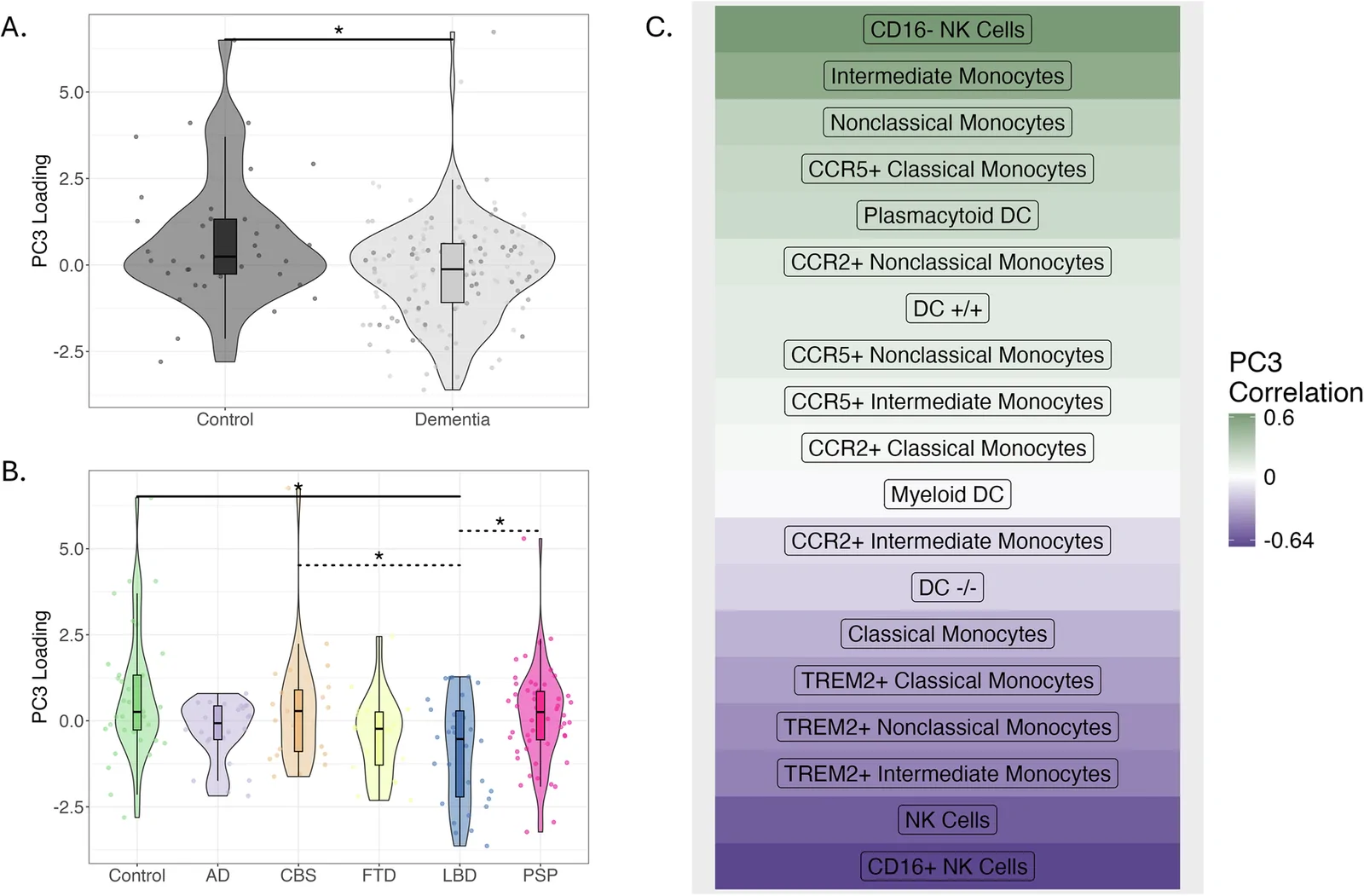
Immune profile loading onto CD16- NK cells (positively) and CD16+ NK cells (negatively). **A** Median individual loading onto PC3 between controls and all-cause dementia patients, W = 2025, *p* = 0.015. **B** Median individual loading onto PC3 for each group. Post hoc comparisons indicate LBD differs individually from controls, (H = 3.38, *p* = 0.015). **C** Cellular correlations to PC3 extracted following PCA. Darker colours indicate stronger positive and negative correlations to PC3. Mann–Whitney U and Kruskal–Wallace tests were used to compare medians followed by Dunn’s Post hoc analysis with FDR correction for multiple comparison. A dashed line indicates a result of statistical significance prior to FDR correction that did not maintain significance following correction.

From the PCA on the 19 cell populations, three components were selected for further analyses. Together, these components accounted for half of the total variance in the data. Supplementary Table S3 shows the contribution of each cell type to the selected components. The first principal component (PC1) accounted for 17.97% of the variance and was strongly positively weighted by TREM2+ monocytes and nonclassical monocytes (excluding CCR5+ nonclassical monocytes). PC1 was most negatively loaded onto classical monocytes, including CCR2+ classical monocytes, as well as NK cells high in CD16 expression (CD16+ NK cells) (Fig. 2C). The second principal component (PC2) accounted for 16.02% of the total variance. PC2 was strongly, negatively weighted by intermediate monocytes and CCR5+ monocytes, and strongly positively weighted by dendritic cells negative for CD11c and CD123 (Fig. 3C). The third principal component (PC3) accounted for 12.98% of the total variance and was most strongly positively weighted by CD16- NK Cells, and most strongly, negatively weighted in CD16+ NK cells and TREM2+ monocytes (Fig. 4C). PC relationships to sex and age are found in Supplementary Table S4. Regression analysis revealed that there was no significant relationship between individual loadings in PC1, PC2, or PC3 and age (*p* >0.05). There was no significant relationship between individual loadings in PC1, PC2, or PC3 and sex (*p* >0.05). We identified 3 patients (2 FTD and 1 CBS) with hsCRP > 10 mg/L. Similar components and individual component loadings were obtained when excluding these participants, which may be indicative of either central or peripheral inflammatory conditions (see Supplementary Fig. S7, Table S5).

All-cause dementia patients showed significantly lower median loading values in PC1 (U = 3351, *p* = 0.035) as compared to controls (Fig. 2A). Multi-group comparison did not identify a significant difference in PC1 loading between controls and each patient group (H = 10.4, *p* = 0.065) (Fig. 2B). There was no statistically significant difference in individual PC2 loading between all-cause dementia and controls (U = 2844, *p* = 0.767) or between controls and each patient group (H = 3.88, *p* = 0.567) (Fig. 3A, B). All-cause dementia patients had significantly reduced PC3 loading as compared to controls (U = 2025, *p* = 0.0145) (Fig. 4A). Multi-group comparison identified a significant difference in PC3 loading across groups (H = 15.9, *p* = 0.007), and post hoc analysis revealed that patients with LBD showed significantly lower median loading values in PC3 than controls (*p*-FDR = 0.01) (Fig. 4B). See Supplementary Table S6 for statistics from post hoc analysis group comparisons.

### Clinical outcomes

Individual patient loadings of PC1, PC2, and PC3 were each evaluated as a predictor of ACE-R scores including the interaction of diagnostic group as an independent variable. Regression analysis did not reveal significant predictive value of PC1, PC2, or PC3 for ACE-R total scores. There was no interaction between diagnostic group, age, or sex, with any principal component in terms of ACE-R score.

In the subset with biomarker data, patient loadings of PC1, PC2, and PC3 were each evaluated as a predictor of plasma-based biomarker values (mg/L) including the interaction of diagnostic group as an independent variable. Regression analysis did not reveal significant predictive value of PC1, PC2, or PC3 for p-tau217, NfL, Aβ40/Aβ42, or GFAP (*p* < 0.05). There was no interaction between diagnostic group, age, or sex, with any principal component in terms of these biomarkers.

We used our data to evaluate the sample size required for traditional Cox proportional hazards regression. We completed a power calculation (power = 0.8, alpha = 0.05). To detect a hazard ratio  <0.8 or >1.2 as seen in previous studies using this regression in immune data in dementia, we would require 234 patients with confirmed death, or 835 total participants given our current distribution of deceased to living patients (28%).

**Fig. 5:**
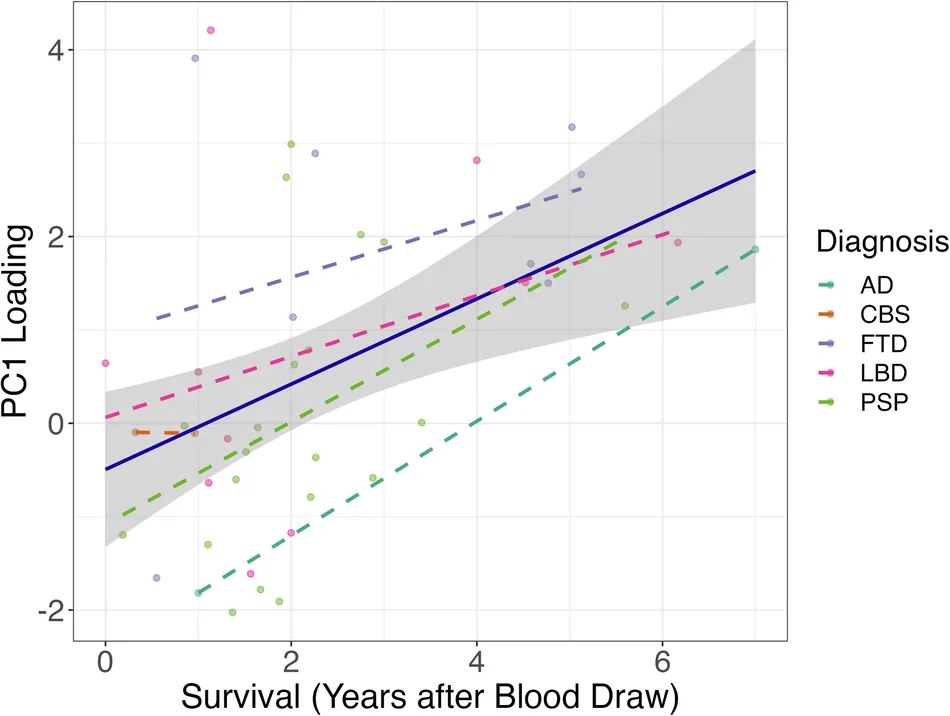
Years of survival following blood draw correlate with individual PC1 scores across diagnostic groups. A linear regression was used to establish how years of survival following blood draw was predicted by individual PC1 loading across all patient groups (β = 0.450, F = 10.098, *p* = 0.0026, r^2^ = 0.184), in a subset of 42 patients who had died by the census date. Outcomes were considered significant at *p* < 0.05.

Thus, to carry out a primary survival analysis, we tested whether PC1, PC2 or PC3 were predictive of years of time to death in the subset of 42 patients who died prior to our census date (2 AD, 2 CBS, 10 LBD, 8 FTD, and 20 PSP). Higher individual loadings in PC1 predicted longer time intervals from the research visit to death (*y* = 0.4463*x* + 2.0915, F = 10.24, *p* = 0.0027, *r*^2^ = 0.184) (Fig. 5). This correlation remained significant after controlling for age, sex, and diagnosis (F = 3.48, *p* = 0.00507, *β*_Age_= −0.025, *β*_Male_=0.13). Importantly, PC1 predicted survival over and above all biomarkers when including NfL, Aβ40/Aβ42, p-tau217, and GFAP as covariates in our model (F = 1.443, *p* = 0.035, *β*NfL= −0.012, *β*p-tau217 =  1.23, *β*AB42/AB40 =   − 0.09, *β*GFAP =  0.001). There was no association between PC2 (F = 0.1043, *p* = 0.75) or PC3 (F = 2.983, *p* = 0.09) and years of survival following blood draw.

## Discussion

The main outcome of this study is confirmation that peripheral blood-based innate immunophenotypes are abnormal in people with AD, LBD, FTD, PSP, and CBS. The principal components of the 19-cell class immunophenotype were similarly abnormal in each type of neurodegenerative disease, and one component (or immune profile) predicted time to death. We identified transdiagnostic similarly in the *magnitude* of this abnormality in PC1, and diagnostic specificity in the magnitude and composition of this abnormality for LBD patients in PC3. Importantly, the multivariate *pattern* of cell-types was dissimilar between controls and all-cause dementia participants in this study. Taken together, these results indicate that even peripheral immune profiles have diagnostic specificity and identify a cellular profile represented by a redistribution of monocyte subtypes as being linked to survival across neurodegenerative dementias.

In the introduction, we proposed that peripheral innate immune cells are directly related to the pathogenesis of neurodegenerative diseases, based on genomics data and the physiological integration between central and peripheral immune compartments [2–7, 38–43, 59, 60]. Rather than a cell-by-cell account, we adopted a multivariate approach and identified three principal components of interest. The first (PC1) was weighted positively to TREM2+ and nonclassical monocytes and negatively to classical monocytes. The second (PC2) was weighted positively to CCR5+ monocytes and intermediate subtypes, and negatively to DC-/- cells. The third component (PC3) was positively weighted by CD16- NK cells and negatively weighted by CD16+ NK cells. These multivariate (multi-cell type) patterns help to contextualize the results of more selective prior studies.

For example, previous work demonstrated altered monocyte subpopulations in AD that varied with disease stage [41], albeit without natural killer cell or dendritic cell data. While we do not present data on disease severity, our data corroborate these findings by suggesting aberrant levels of monocyte subtypes in neurodegenerative disease. In addition, monocyte subtype redistribution may explain why total monocytes have not been found to be different in the MCI stage as compared to controls [42]. Similarly, our identification of greater CD16+ NK cell overexpression in disease adds to existing literature identifying NK cell alterations in AD versus controls, however, this study did not compare CD16+ and CD16- NK cell subtypes [43]. Peripheral myeloid DCs have previously been found to be altered in AD and Parkinson’s disease versus controls [38, 39]. While results from PC2 suggest AD may indeed be characterized by a trend of reduced myeloid DCs (Fig. 3B), these cells did not strongly correlate to PC1 or PC3, thus underscoring the potential relative importance of monocytes and NK cells in all-cause neurodegenerative dementia. In all, our work builds upon extant immunophenotyping literature and confirms monocyte and NK cell population changes with multiple neurodegenerative diseases.

Monocytes are a heterogeneous population of innate immune cells [61]. They have diverse functions: cytokine release, migration to damaged tissues, and differentiation into phagocytic cells. Based on surface markers, monocytes can be divided into classical, nonclassical, and intermediate subtypes. Classical monocytes are pro-inflammatory, potentially neurotoxic, and are recruited to damaged tissue during inflammation [62]. Nonclassical monocytes are anti-inflammatory and may be neuroprotective [63, 64]. In addition, monocytes may express functional receptors such as CCR2, a chemotaxis receptor controlling the recruitment of the cell, or TREM2, a receptor controlling phagocytic and inflammatory function [65, 66]. In contrast, NK cells in other diseases facilitate the death of infected cells, regulate the adaptive immune response through cytokine production, and mediate autoimmunity. NK cells can be subdivided based on their relative presentation of surface markers into CD16-, regulating cytokine production, or CD16+, indicating cytotoxicity [67]. NK cells have been implicated in many disorders of the central nervous system, including neurodegenerative dementia [43, 68, 69]. Finally, dendritic cells are monocyte-derived antigen presenting immune cells that have been linked to Parkinson’s disease and AD [38, 39].

Although previous evidence suggested monocyte, NK, and dendritic cells are each involved in neurodegenerative diseases, prior evidence was mainly gleaned from investigation in Alzheimer’s disease. Our results identify abnormal features of the peripheral innate immune system that are common across AD, LBD, FTD, CBS and PSP. By using data-driven analytic methods, we explore multivariate peripheral features that may not be revealed by evaluating one cellular type at a time. Innate immune patterns across the 19 cells investigated in this study identify patient groups to be dissimilar from controls and exhibit a cluster of innate immune patterns in FTLD as compared to LBD and AD. Interestingly, we confirmed cases of amyloid pathology in CBS, AD, and LBD patients. A relatively smaller distance between CBS and AD/LBD as compared to PSP and FTD suggests a shared innate immune pattern, at least in part, may reflect amyloid pathology (Fig. 1). Through the application of dimensionality reduction techniques, our results imply neurodegenerative disease to be characterized by pro-inflammatory and cytotoxic peripheral immune dynamics atypical to healthy aging.

We observed reduced PC1 loading in all-cause dementia patients versus controls (Fig. 2A). This suggests that reduced relative expression of TREM2+ on peripheral monocytes and increased relative prevalence of classical monocytes may be a characteristic pro-inflammatory and cytotoxic immune signature linked to neurodegenerative dementia. TREM2 is involved in modulating inflammation, mediating phagocytosis, and promoting myeloid cell survival [65, 70]. While the current study did not evaluate CSF levels of sTREM or CCL2, looking at TREM2 and CCR2 expressed on peripheral monocytes enables the investigation of functional dynamics of active peripheral cells accessible from blood. In the context of neurodegenerative diseases, TREM2 expressed on microglia is linked to prevention or downregulation of tau hyperphosphorylation and cited to harbour a protective effect in neurodegenerative disease [71, 72]. However, this interaction has been investigated primarily in microglia of murine models of AD rather than peripheral monocytes and human studies across the dementia spectrum. Our results support claims of TREM2 as an important receptor acting from the periphery in neurodegenerative disease [27]. While TREM2 is often reported in relation to microglia, transcriptional analyses in the human brain suggest the infiltration of TREM2 expressing monocyte-derived immune cells in patients with AD [73]. In addition, elevated levels of TREM2 transcript in peripheral blood cells is found to be protective in the clinical progression of AD [74]. Although the current study is correlational without evidence of causality, we build upon the clinical relevance of peripheral TREM2 by analysing peripheral monocyte dynamics, rather than TREM2 expressing cells alone.

In contrast to the diagnostic generalizability of PC1, patients with LBD displayed lower median individual loadings in PC3 versus controls (Fig. 4B). This third component was weighted positively by CD16- NK cells and negatively by NK Cells and CD16+ NK cells. Given CD16+ NK cells contributed to the negative arm of both PC1 and PC3, this cell type is of interest in all-cause dementia. However, LBD was particularly characterized by an increased number in NK cells and a functional shift in NK cell subtypes. Indeed, human postmortem studies show redistribution of NK cellular subtypes in patients with alpha-synuclein pathology, suggesting a potentially strong effect in this disease group [37, 75]. Beyond this, reduced average PC3 loading in LBD suggests this cohort may harbour TREM2 dynamics that are dissimilar to other dementias as these cells comprise the negative end of PC3 (opposite to PC1 composition). The present findings distinguishing LBD are in line with previous observed differences in microglial activation and TREM2 genetic relationships in LBD as compared to other diseases [18, 29, 76]. However, because we did not identify relationships between PC3 and clinical or cognitive outcomes, the relevance of NK cells in therapeutic target and monitoring of LBD patients must be further investigated.

The results presented in this study are relevant to therapeutic development and application in neurodegenerative disease. We identify inflammatory profiles that are transdiagnostic and prognostic, pointing toward the application of successful inflammatory therapeutics across neurodegenerative diseases. As there are an increasing number of immunomodulatory drugs under evaluation in dementia [77, 78], there is an increasing need to understand immune changes in neurodegeneration so that these therapies may be accurately targeted and monitored with appropriate biomarkers of response in clinical trials. This is particularly pertinent for our understanding of the functional outcomes associated with TREM2, where animal models show conflicting results [33]. Further studies are needed to clarify the functional role of TREM2 as expressed by different cells of the immune system in humans [73]. Specifically, our results probe further investigation into common features of immune profiles for the effective adaptation of treatment and disease monitoring.

The clinical relevance of shifting peripheral monocyte patterns toward a protective, anti-inflammatory phenotype is highlighted by the associations of PC1 individual loading with survival across patient groups (Fig. 5). Increased peripheral TREM2+ expression and nonclassical monocytes combined with reductions in classical monocytes and CD16+ NK cells may represent a protective effect in neurodegenerative disease, while the opposite patterns may contribute to accelerated clinical decline. Our survival analysis revealed PC1 as a strong predictor of survival over and above plasma dementia-relevant markers. This result suggests that the cell types dominating PC1 may harbour important prognostic monitoring information to be applied to clinical practice and clinical trials.

While further investigation is certainly required to establish effective PBMC biomarkers for clinical use, akin to ongoing investigations in lymphocyte/monocyte ratios [79], dynamic changes in monocyte composition (TREM2+ and classical/nonclassical monocytes) may serve as an accessible, transdiagnostic node for dementia monitoring and prediction. Moreover, the present link between increased TREM2+ monocytes and prognosis is especially relevant in light of ongoing clinical trials targeting TREM2 in people with AD [80]. Given the transdiagnostic loading of PC1, TREM2 mediated treatments may be effective in other neurodegenerative conditions. Because we did not identify any relationships between immune profiles and biomarkers of cerebral pathology measured in a subset of our participants, our results do not suggest monocyte redistribution to be a strong diagnostic or pathological biomarker for dementia, but rather may enable transdiagnostic enrichment of clinical trials and practice.

Our study has several limitations. First, patient recruitment and cohort definition are based on clinical criteria rather than pathology-confirmed cases, although clinic-pathological correlations are high for PSP, FTD, LBD and AD. Next, the heterogeneity within cohorts (e.g., grouping bvFTD with PPA) and co-pathologies (e.g., CBS underpinned by CBD and/or AD) may complicate the interpretation of the results and reduce sensitivity to between-group differences. In addition, group sizes are unbalanced, although the non-parametric tests used are relatively robust to moderate variation in group size. Although the present cohort is representative of patients who are referred in dementia clinics, future studies with larger sample sizes with more pathology-specific markers and genotyping (including APOE) may be able to clarify the interaction between innate immune system and co-pathologies across all syndromes. In addition, our cohort was predominantly white/caucasian reflecting the ethnicity distribution of the over 65-year-old population in the UK. Further studies are needed in ethnically diverse populations, to test the generalisation of our results. Importantly, ACE-R was used as the primary assessment to capture cognitive decline as this exam is widely implemented in memory clinics. However, this screening-test varies in sensitivity to the domain-specific cognitive changes associated with different diagnoses, and this may have reduced power to detect correlations with cognitive deficits. Additionally, presently investigated peripheral cell types are not dementia-specific and may capture comorbidities that are not directly linked to dementia. We have attempted to mitigate the potential confound of co-occurring inflammatory conditions, or the use of specific anti-inflammatory medication in several ways. First, the source study excluded patients with co-morbid pro-inflammatory conditions, such as rheumatoid arthritis, inflammatory bowel disease, psoriasis or other autoimmune disorders, and cancer; and all contributory cohorts excluded recent systemic infections and current acute medical illness. Moreover, we demonstrate that exclusion of the small number of patients with elevated hsCRP, indicative of systemic inflammation, does not alter the main results of the study (Supplementary Fig. S7). In this this study, we were unable to include correlations with central measures of inflammation, as imaging was not a requirement for recruitment and participant inclusion. However, recent studies have shown strong associations between blood-based inflammatory markers (i.e. cytokines) and central inflammation in dementia, as measured by TSPO PET [81]. Linking innate immune blood-based profiles with central inflammation will be a key step for future research. Finally, data collection spanned several years, which may have led to batch variation in our immunophenotyping analysis. To help control sample variability, sample gating was conducted by a single person, and validated against the gating of two other experts on a sub-sample.

In conclusion, the present study provides a comprehensive characterization of the peripheral innate immune system in multiple neurodegenerative dementias. We suggest dysfunctional patterns of the innate immune system are characteristic of neurodegenerative diseases. Blood-derived innate immune profiles can distinguish sub-populations of cells relevant to diverse clinical cohorts and their prognosis. Further studies are needed to clarify interactions between the peripheral innate immunity profiles and dementia-related events in the cerebrum, including neuroinflammation. We hope that the identification of blood-based innate immune profiles can contribute to enhanced precision medicine approaches dementia, to identify new therapeutic targets and improve clinical trial design for immunotherapies.

## Supplementary information

Supplementary figures and tables

## Data Availability

Anonymized processed data can be shared upon request to the corresponding author. Raw data may also be requested but are likely to be subject to a data transfer agreement with restrictions required to comply with participant consent and data protection regulations.
