# Supplementary material for: Peripheral innate immunophenotype in neurodegenerative disease: blood-based profiles and links to survival
Source: Mol Psychiatry. 2024 Oct 29;30(5):1985–94. doi: 10.1038/s41380-024-02809-w (PMC12015116; doi:10.1038/s41380-024-02809-w)
Supplement: Supplementary file 1 — Supplementary figures and tables [file 41380_2024_2809_MOESM1_ESM.docx]

**Peripheral innate immunophenotype in neurodegenerative disease: blood-based profiles and links to survival**

Alexandra Strauss^1^, Peter Swann^2^, Stacey L Kigar^2,3^, Rafailia Christou^1^, Natalia Savinykh Yarkoni^3^, Lorinda Turner^2,3^, Alexander G Murley^1^, Leonidas Chouliaras^2^, Noah Shapiro^1^, Nicholas J Ashton^4,5,6^, George Savulich^2^, W Richard Bevan-Jones^2^, Ajenthan Surendranthan^2^, Kaj Blennow ^4,7^, Henrik Zetterberg ^4,7,8,9,10,11^, John T O’Brien^2^, James B Rowe^1,12^,

Maura Malpetti^1,13^

1 University of Cambridge Department of Clinical Neurosciences and Cambridge University Hospitals NHS Trust, Cambridge, United Kingdom

2 Department of Psychiatry, University of Cambridge, Cambridge, United Kingdom

3 Department of Medicine, University Cambridge, Cambridge, United Kingdom

4 Department of Psychiatry and Neurochemistry, University of Gothenburg, Gothenburg, Sweden

5 Banner Alzheimer's Institute and University of Arizona, Phoenix, AZ, USA

6 Banner Sun Health Research Institute, Sun City, AZ 85351, USA5

7 Clinical Neurochemistry Laboratory, Sahlgrenska University Hospital, Mölndal, Sweden

8 Department of Neurodegenerative Disease, UCL Institute of Neurology, Queen Square, London, UK

9 UK Dementia Research Institute at UCL, London, UK

10 Hong Kong Center for Neurodegenerative Diseases, Clear Water Bay, Hong Kong, China

11 Wisconsin Alzheimer’s Disease Research Center, University of Wisconsin School of Medicine and Public Health, University of Wisconsin-Madison, Madison, WI, USA

12 Medical Research Council Cognition and Brain Sciences Unit, Cambridge, United Kingdom

13 UK Dementia Research Institute at University of Cambridge, Cambridge CB2 0XY, UK

Corresponding Author:

Dr. Maura Malpetti

Department of Clinical Neurosciences

University of Cambridge

Herchel Smith Building, Forvie Site Robinson Way,

Cambridge Biomedical Campus Cambridge

CB2 0SZ

Email: mm2243@medschl.cam.ac.uk

# Supplementary Figures

*Figure S1. Graphic showing cell types in the study. Red arrows indicate the parent population that each raw cell below was divided by to yield the ratio value used for analysis. Grey populations were not considered in this analysis.*


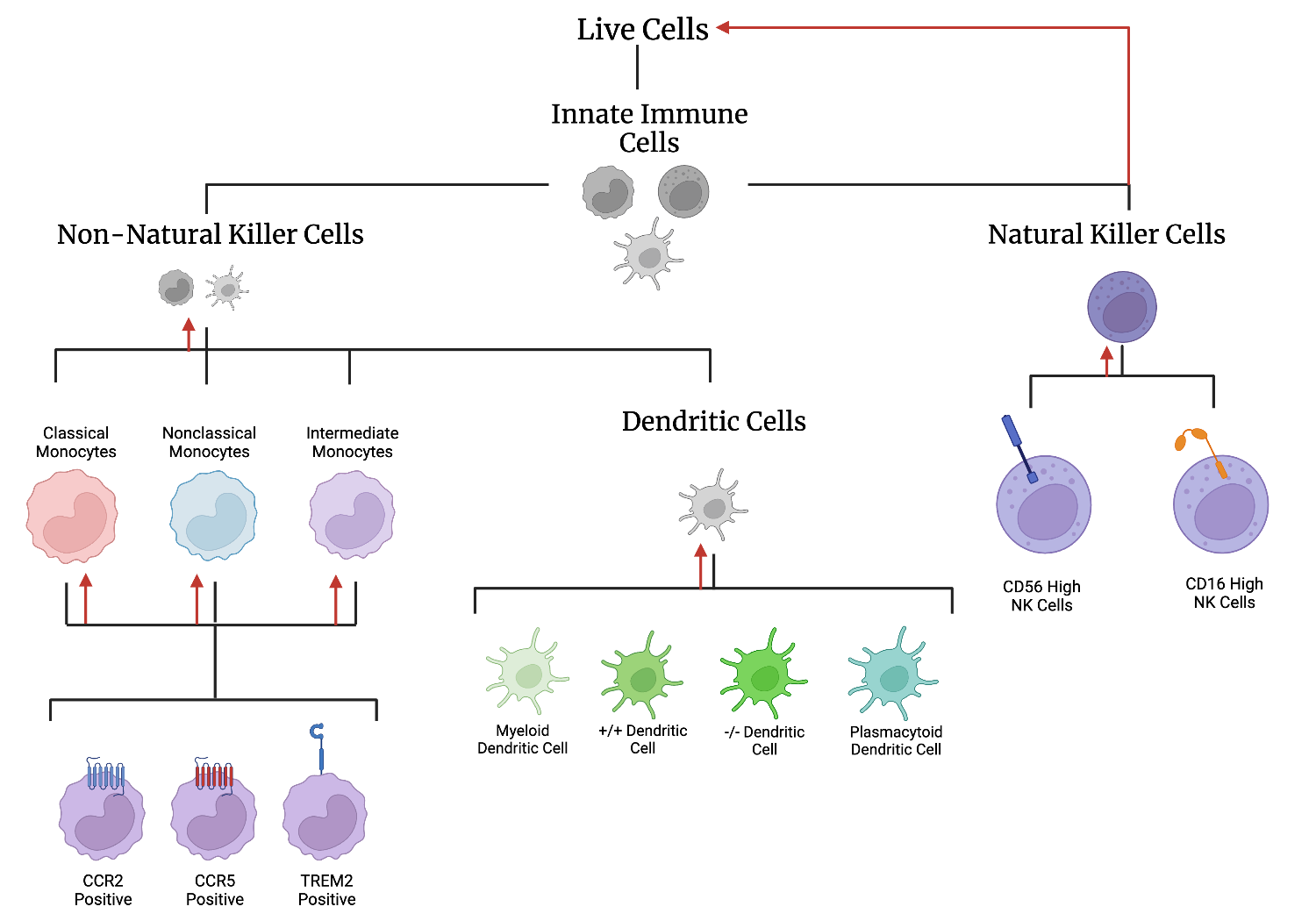


*Figure S2. Gating Strategy for Flow Cytometry analysis. Samples were first evaluated for compliance by gating a continuous column flow over time. Next, all live cells were sectioned. B and T cells were eliminated by selecting cells negative for CD3, CD19, and CD20.*


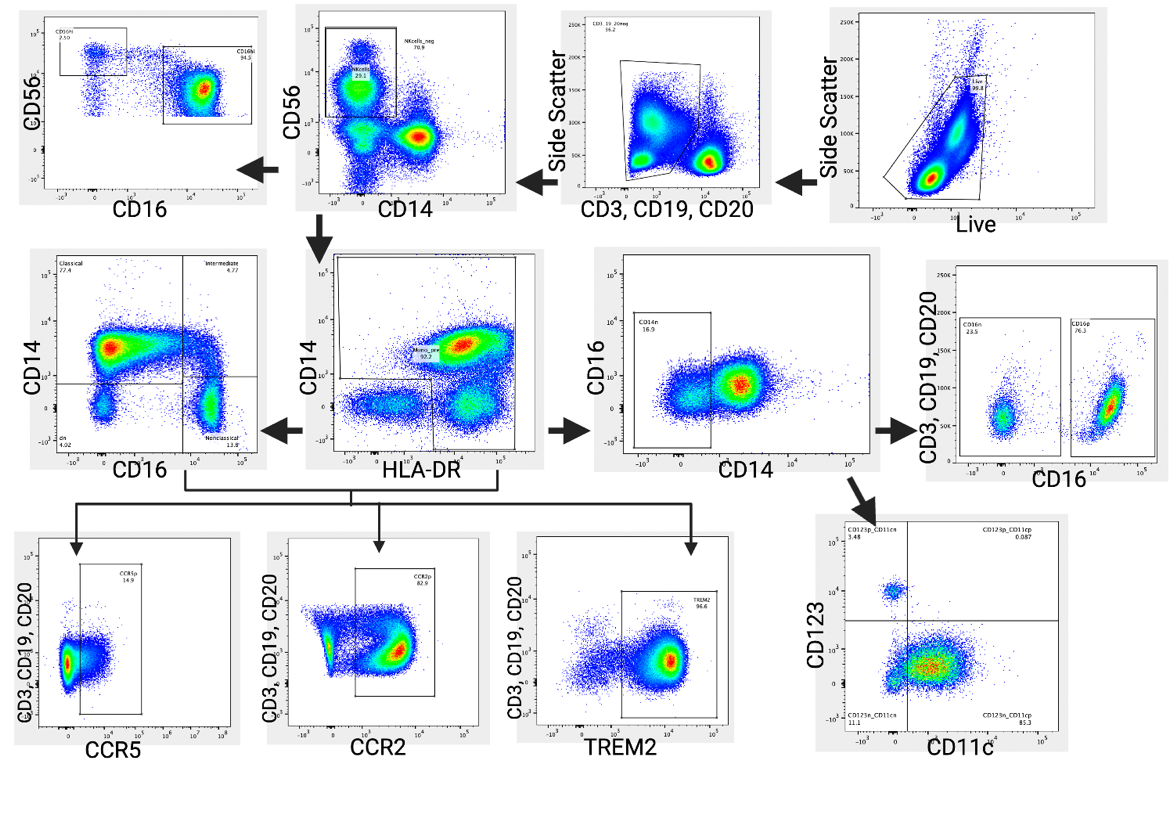

*Figure S3. Visual Verification of Gating Strategy. Ten patients were selected and gated by three distinct raters. In addition to ICC analysis, gates that were visually inconsistent were revisited and the strategy was agreed upon between the three raters before continuing with analysis. (e.g. Gater 3 had consistently different values past figure S3.23 which were addressed).*

###

*Figure S4. Scree plot from PCA computed following the removal of outliers.*
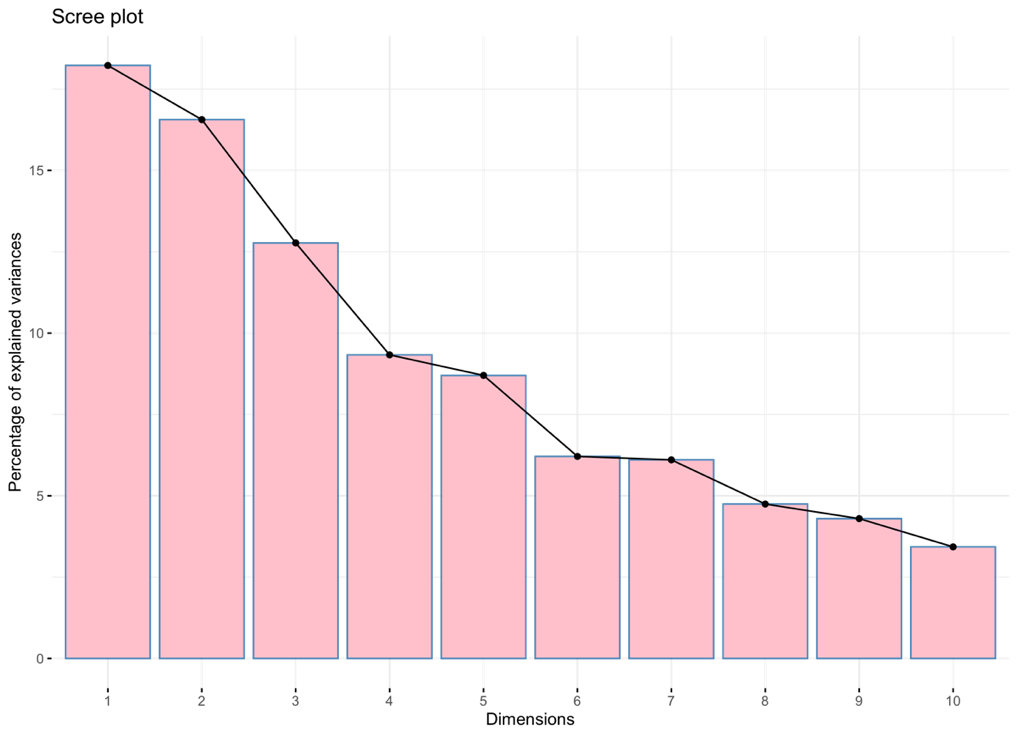


*Figure S5. Median prevalence of innate immune cells (as a ratio of parent population) between dementia patients and controls. * indicates statistical significance following FDR correction, + indicated statistical significance prior to FDR correction.*

*Figure S6. Median prevalence of innate immune cells (as a ratio of parent population) between AD, FTD, PSP, LBD, CBS, and controls. * indicates statistical significance following FDR correction, + indicated statistical significance prior to FDR correction.*

*Figure S7. Comparing component outputs following removal of participants with hsCRP >10mg/L. Innate immune cell-derived principal components (PC) and correlation on individual scores obtained from the full cohort (n=185) and the reduced cohort (n=182) after excluding participants with confirmed hsCRP > 10 mg/L.*

# Supplementary Tables

*Table S1. Antibodies, blocking reagent, and viability marker used in this study*

| **Antibody** | **Vendor** | **Catalogue** |
| --- | --- | --- |
| CD56-FITC | eBioscience | 11-0566-42 |
| CD123-PerCP/Cy5.5 | eBioscience | 45-1239-42 |
| TREM2-PE  or  Rat IgG2B-PE (iso) | R&D Systems | FAB17291P  or  IC013P |
| CD11c-PE/Vio770 | Miltenyi Biotec | 130-113-581 |
| CCR5-AlexaFluor647 | eBioscience | 313712 |
| CD20-APC/Cy7 | eBioscience | 47-0209-42 |
| CD19-APC/Cy7 | eBioscience | 47-0199-42 |
| CD3-APC/Cy7 | BioLegend | 317342 |
| CCR2-BV421 | BioLegend | 357210 |
| CD14-BV605 | BioLegend | 301834 |
| CD16-BV650 | BioLegend | 302042 |
| HLADR-BV785 | BioLegend | 307642 |
| Live/Dead Aqua | Thermo Fisher | L34957 |

*Table S2. ICC Results across major gates. The gating strategy was validated between three distinct raters using the same gating strategy individually for 10 shared samples. Inter-rater correlation was assessed graphically as well as mathematically using intraclass correlation coefficient (ICC) analysis: ICC 2,1 (Shrout and Fleiss, 1979). Classical Monocyte and Nonclassical Monocyte inconsistencies were evaluated and normalized prior to continuing with the analysis. The ICC value upon adjustment is also reported.*

| Gate | ICC Value |
| --- | --- |
| Live | 0.96 |
| Innate Cells | 0.96 |
| Natural Killer Cells | 0.95 |
| Non-Natural Killer Cells | 0.97 |
| Dendritic Cells | 0.95 |
| Classical Monocytes | 0.54 -> 0.91 |
| Intermediate Monocytes | 0.9 |
| Nonclassical Monocytes | 0.12 -> 0.9 |

*Table S3. Individual cell type percent contribution to principal component (PC) 1, 2, and 3.*

| Cell Type | PC1 | PC2 | PC3 |
| --- | --- | --- | --- |
| TREM2+ Classical Monocytes | 17.55 | 1.27 | 4.95 |
| TREM2+ Nonclassical Monocytes | 17.07 | 2.79 | 7.03 |
| TREM2+ Intermediate Monocytes | 14.99 | 3.51 | 8.52 |
| Classical Monocytes | 11.39 | 1.31 | 3.18 |
| Nonclassical Monocytes | 9.16 | 0.25 | 3.63 |
| CCR2+ Nonclassical Monocytes | 7.68 | 1.49 | 0.94 |
| CCR2+ Classical Monocytes | 4.88 | 10.93 | 0.09 |
| CD16+ NK Cells | 4.77 | 0.28 | 20.19 |
| DC +/+ | 3.58 | 0.28 | 0.69 |
| DC -/- | 3.46 | 13.03 | 1.14 |
| CCR5+ Classical Monocytes | 1.83 | 3.90 | 2.80 |
| Intermediate Monocytes | 0.97 | 0.24 | 11.22 |
| CCR2+ Intermediate Monocytes | 0.86 | 15.10 | 0.74 |
| CD16- NK Cells | 0.78 | 0.00 | 16.30 |
| CCR5p Intermediate Monocytes | 0.43 | 17.27 | 0.27 |
| NK Cells | 0.32 | 0.05 | 15.20 |
| Plasmacytoid DC | 0.12 | 0.37 | 2.39 |
| Myeloid DC | 0.10 | 12.34 | 0.02 |
| CCR5+ Nonclassical Monocytes | 0.06 | 15.62 | 0.69 |

*NK = Natural Killer, TREM = Triggering Receptor Expressed on Myeloid Cells, DC = Dendritic Cells*

*Table S4. Results for principal component relationships to Age, Sex, and ACE-R score*

|  | PC1 | PC2 | PC3 |
| --- | --- | --- | --- |
| Age Interaction Statistics  Statistic, p-value | f= 0.099,  p = 0.753 | f= 0.1381,  p= 0.711 | f= 0.5979,  p= 0.440 |
| Sex Interaction Statistics  Statistic, p-value | U=3739,  p = 0.281 | U= 4243,  p = 0.742 | U= 3598,  p = 0.141 |
| ACE-R Correlation Statistics  statistic, p-value | f= 0.6024,  p =0.439 | f =0.1903,  p = 0.663 | f= 0.853,  p= 0.357 |

*Table S5. PCA analysis with and without participants with high hsCRP. In a subset of participants (Controls = 10, AD = 1, CBS = 6, FTD = 6, LBD = 15, and PSP = 16), serum was analyzed at the Core Biochemistry Assay Laboratory of Cambridge University Hospitals NHS Foundation Trust. The assay for the high sensitivity c-reactive protein (hsCRP) was carried out using Siemens Dimension EXL autoanalyzer. We identified 3 patients (2 FTD and 1 CBS) with hsCRP >10 mg/L. We recomputed the PCA excluding these 3 participants, the PCA results did not change.*

| Cell Order | PC1-  Original | PC2-  Original | PC3-  Original | Cell Order | PC1- excluding hsCRP > 10 | PC2- excluding hsCRP > 10 | PC3- excluding hsCRP > 10 |
| --- | --- | --- | --- | --- | --- | --- | --- |
| Classical TREM2+ | 0.78 | 0.17 | 0.35 | Classical TREM2+ | 0.78 | 0.16 | 0.34 |
| Nonclassical TREM2+ | 0.77 | 0.27 | 0.42 | Nonclassical TREM2+ | 0.78 | 0.26 | 0.41 |
| Intermediate TREM2+ | 0.72 | 0.30 | 0.46 | Intermediate TREM2+ | 0.73 | 0.30 | 0.44 |
| Nonclassical Monocytes | 0.56 | -0.11 | -0.29 | Nonclassical Monocytes | 0.56 | -0.17 | -0.25 |
| Nonclassical CCR2+ | 0.52 | 0.20 | -0.15 | Nonclassical CCR2+ | 0.55 | 0.13 | -0.11 |
| Classical CCR5 + | 0.31 | -0.65 | 0.17 | Classical CCR5 + | 0.32 | 0.29 | -0.25 |
| DC -/- | 0.26 | 0.34 | -0.26 | DC -/- | 0.25 | -0.65 | 0.23 |
| Intermediate Monocytes | 0.19 | 0.09 | -0.52 | Intermediate Monocytes | 0.23 | 0.03 | -0.52 |
| CD16- NK Cell | 0.17 | 0.00 | -0.63 | CD16- NK Cell | 0.18 | -0.06 | -0.64 |
| CCR5+ Nonclassical Monocytes | 0.07 | 0.69 | -0.13 | CCR5+ Nonclassical Monocytes | 0.12 | 0.67 | -0.18 |
| Myeloid DC | -0.03 | 0.62 | 0.02 | Myeloid DC | 0.00 | 0.61 | -0.03 |
| Plasmacytoid DC | -0.06 | -0.09 | -0.24 | CCR5+ Intermediate Monocytes | -0.05 | 0.72 | -0.14 |
| CCR5+ Intermediate Monocytes | -0.10 | 0.73 | -0.08 | Plasmacytoid DC | -0.06 | -0.11 | -0.25 |
| CCR2+ Intermediate Monocytes | -0.11 | -0.04 | 0.61 | CCR2+ Intermediate Monocytes | -0.09 | 0.70 | 0.12 |
| NK Cells | -0.15 | 0.68 | 0.14 | NK Cells | -0.13 | 0.02 | 0.61 |
| DC +/+ | -0.34 | 0.11 | -0.13 | DC +/+ | -0.32 | 0.13 | -0.14 |
| CCR2+ Classical Monocytes | -0.39 | 0.59 | -0.05 | CCR2+ Classical Monocytes | -0.34 | 0.61 | -0.08 |
| CD16 + NK Cell | -0.41 | -0.08 | 0.70 | CD16 + NK Cell | -0.43 | 0.00 | 0.70 |
| Classical Monocytes | -0.62 | 0.22 | 0.28 | Classical Monocytes | -0.62 | 0.29 | 0.21 |

*NK = Natural Killer, TREM = Triggering Receptor Expressed on Myeloid Cells, DC = Dendritic Cells*

*Table S6. Results for Dunn’s Post hoc analysis in individual PC3 loading. Results for comparisons between each group individual loading in PC3in the three components selected. * Indicates significance at p< 0.05.*

|  |  | Statistic | P Value | P Value- FDR Corrected |
| --- | --- | --- | --- | --- |
| Control | AD | 2.10 | 0.04* | 0.426 |
| Control | CBS | 0.77 | 0.44 | 1 |
| Control | LBD | 2.38 | 0.212 | 0.01* |
| Control | FTD | 2.41 | 0.0163 | 0.212 |
| Control | PSP | 1.03 | 0.31 | 1 |
| AD | CBS | -1.18 | 0.245 | 1 |
| AD | LBD | 1.05 | 0.286 | 1 |
| AD | FTD | 0.459 | 0.634 | 1 |
| AD | PSP | -1.35 | 0.185 | 1 |
| CBS | LBD | 2.27 | 0.0228 | 0.273 |
| CBS | FTD | 1.55 | 0.121 | 1 |
| CBS | PSP | 0.051 | 0.957 | 1 |
| LBD | FTD | 0.487 | 0.63 | 1 |
| LBD | PSP | -2.69 | 0.00668 | 0.094 |
| FTD | PSP | -1.74 | 0.0816 | 0.816 |

*AD = Alzheimer’s disease, LBD= Lewy Body disease, PSP= Progressive Supranuclear Palsy, FTD = Frontotemporal Dementia*

*Table S7. Results for pairwise comparisons of each cell type between dementia patients and controls. * Indicate significance at p< 0.05. See Figure S6 for graphical representation of values.*

| Cell Type | Statistic | P Value | P Value- FDR Corrected |
| --- | --- | --- | --- |
| CCR2+ Classical Monocytes | 2265 | 0.09 | 1 |
| CCR5+ Classical Monocytes | 2541 | 0.46 | 1 |
| TREM2+ Classical Monocytes | 2847 | 0.75 | 1 |
| CCR2+ Intermediate Monocytes | 2778 | 0.94 | 1 |
| CCR5+ Intermediate Monocytes | 2745 | 0.97 | 1 |
| TREM2+ Intermediate Monocytes | 2958 | 0.49 | 1 |
| CCR2+ Nonclassical Monocytes | 3857.5 | 1.74E-04** | 0.003306* |
| CCR5+ Nonclassical Monocytes | 2741.5 | 0.96 | 1 |
| TREM2+ Nonclassical Monocytes | 3016 | 0.37 | 1 |
| DC-/- | 2853 | 0.74 | 1 |
| Myeloid DC | 2711 | 0.87 | 1 |
| Plasmacytoid DC | 3120 | 0.21 | 1 |
| DC+/+ | 2695 | 0.83 | 1 |
| CD16+ NK Cells | 2184 | 0.05 | 0.969 |
| CD16- NK Cells | 3437 | 0.020* | 0.3857 |
| Classical Monocytes | 1805 | 0.0012** | 0.02242* |
| Intermediate Monocytes | 3075 | 0.27 | 1 |
| Nonclassical Monocytes | 3697 | 0.0013** | 0.02546* |
| NK Cells | 1988 | 0.0088** | 0.16701 |

*NK = Natural Killer, TREM = Triggering Receptor Expressed on Myeloid Cells, DC = Dendritic Cells*

*Table S8. Results for Kruskal Wallace comparisons of each cell type between FTD, LBD, PSP, CBS, AD, and Controls. * Indicate significance at p< 0.05. See Figure S7 for graphical representation of values.*

| Cell Type | Statistic | P value | P FDR corrected | Post-hoc group difference |
| --- | --- | --- | --- | --- |
| CCR2+ Classical Monocytes | 10.41 | 0.06 | 1 |  |
| CCR5+ Classical Monocytes | 7.75 | 0.17 | 1 |  |
| TREM2+ Classical Monocytes | 10.59 | 0.06 | 1 |  |
| CCR2+ Intermediate Monocytes | 3.14 | 0.67 | 1 |  |
| CCR5+ Intermediate Monocytes | 3.70 | 0.59 | 1 |  |
| TREM2+ Intermediate Monocytes | 8.40 | 0.13 | 1 |  |
| CCR2+ Nonclassical Monocytes | 23.28 | 2.99E-04 | 0.005 ** | Control > AD (p = 0.01), CBS (p =0.03),  FTD (p = 0.005), PSP (p = 0.01) |
| CCR5+ Nonclassical Monocytes | 4.54 | 0.47 | 1 |  |
| TREM2+ Nonclassical Monocytes | 10.35 | 0.06 | 1 |  |
| DC -/- | 13.75 | 0.017 | 0.32 | FTD >LBD (p =0.031) |
| Myeloid DC | 12.55 | 0.027 | 0.53 | CBS > LBD (p = 0.03) |
| Plasmacytoid DC | 9.57 | 0.088 | 1 |  |
| DC +/+ | 1.31 | 0.93 | 1 |  |
| CD16+ NK Cells | 9.67 | 0.08 | 1 |  |
| CD16- NK Cells | 17.60 | 0.003 | 0.06 | Control > LBD (p =0.002), CBS > LBD ( p= 0.03), PSP > LBD (p =0.039) |
| Classical Monocytes | 20.19 | 0.00115 | 0.02185 * | AD > Control (p < 0.001) |
| Intermediate Monocytes | 15.21 | 0.0094 | 0.18031 | n.s. |
| Nonclassical Monocytes | 19.69 | 0.0014 | 0.02717 * | Control > AD (p < 0.0.001) |
| NK Cells | 12.43 | 0.029 | 0.5586 | LBD > Control (p = 0. 02) |

*AD = Alzheimer’s disease, LBD= Lewy Body disease, PSP= Progressive Supranuclear Palsy, FTD = Frontotemporal Dementia, NK = Natural Killer*
